# A reduced order pseudochannel model accounting for flow maldistribution in automotive catalysis

**DOI:** 10.1038/s41598-025-89756-w

**Published:** 2025-02-11

**Authors:** Pratheeba Chanda Nagarajan, Henrik Ström, Jonas Sjöblom

**Affiliations:** 1 Division of Energy Conversion and Propulsion Systems, Department of Mechanics and Maritime Sciences, Chalmers University of Technology, Gothenburg, 41296 Sweden; 2 Division of Fluid Dynamics, Department of Mechanics and Maritime Sciences, Chalmers University of Technology, Gothenburg, 41296 Sweden; 3 Division of Transport, Energy and Environment, Department of Mechanics and Maritime Sciences, Chalmers University of Technology, Gothenburg, 41296 Sweden

**Keywords:** Catalytic converter, 3D-CFD, 1D-SCM, Pseudochannel, Reduced order model, Chemical engineering, Mechanical engineering, Fossil fuels

## Abstract

Exhaust aftertreatment systems (EATS) play a critical role in reducing emissions and ensuring compliance with stringent emission regulations. Catalytic converters, as part of EATS, involve complex physico-chemical processes. To accurately predict their behavior in realistic geometries, transient 3D models are necessary. However, the computational cost associated with simulations based on such models prevents their application to long-time behaviors as well as in real-time control and diagnostics. While single-channel models (SCMs) are computationally efficient, they struggle to provide accurate predictions during real-time operations with flow maldistribution. In this study, we propose a pseudochannel model derived using steady-state reactive 3D simulations and a nonlinear least squares optimization technique. We show that the performance of this pseudochannel model is superior to a conventional SCM in both transient and steady state test cases. At the same time, the computational cost of the pseudochannel model is equivalent to that of the SCM. These results imply that flow maldistribution effects can be well incorporated in SCMs via a pseudochannel approach that relies on relatively inexpensive steady-state system data.

## Introduction

Nearly two-thirds of the total energy supplied globally is provided by the combustion of natural gas, hydrocarbon oils and biofuels. These energy carriers are responsible for emissions that account for slightly over half of the total greenhouse gas (GHG) emissions^1^. Internal combustion engines account for 98% of transport, and road transport accounts for 23.1% of the total GHG emissions in 2022^2^. Gases such as carbon monoxide (CO), carbon dioxide ($\hbox {CO}_{2}$), hydrocarbons (HC), and nitrogen oxides (${\hbox {NO}_\textrm{x}}$) are emitted in the exhausts from road transport vehicles such as cars, trucks, buses, etc.^3^. Specifically in the European Union (EU) region, of the 903.3 million metric tonnes of $\hbox {CO}_{2}$ emitted in 2022^4^, 15% and 6% were the contributions by light commercial vehicles (LCV) and heavy duty vehicles (HDV) respectively. $\hbox {NO}_\textrm{x}$ emissions have come down to 2.6 million metric tonnes in 2022 and these are significantly reduced from the levels in 1990s (8.1 million metric tonnes)^5^. Automotive exhausts are known to be harmful to human life and the environment. Due to the ubiquitous presence of vehicles in enabling the transportation of people and goods, replacing them and the fuels that power them presents a significant challenge^3^. Alternate fuels that are either carbon neutral or sourced from biomass may reduce the impact of emissions on the environment. Since combustion is exothermic, a large amount of heat is also released during the process. This heat is a primary contributor to the formation of nitrogen oxides ($\hbox {NO}_\textrm{x}$) even in carbon-zero fuels such as hydrogen ($\hbox {H}_{2}$)^6^.

Emission legislations are regulatory frameworks established by governments to control and reduce the release of harmful pollutants into the atmosphere^7^. Over time, emission legislations have become increasingly stringent, reflecting the necessity to counteract the harmful effects on the environment and human life. These emission legislations typically specify emission standards, which are legally enforceable limits on the concentration or mass of pollutants that can be emitted from a specific source. These standards may vary depending on the type of pollutant, the source of emissions, and the geographic region. Common pollutants regulated by emission legislations include nitrogen oxides ($\hbox {NO}_\textrm{x}$), sulfur dioxide ($\hbox {SO}_{2}$), carbon monoxide (CO), unburnt hydrocarbons (UHC), particulate matter (PM). Catalytic converters are used in vehicles to be compliant with emission legislations.

The catalytic converters used in combustion systems such as power plants, stationary engines, automobiles and marine vessels convert some of the aforementioned emissions to $\hbox {CO}_{2}$ and $\hbox {N}_{2}$. These converters are often monolithic reactors having walls coated with a porous washcoat layer that contains the active catalytic materials, such as the platinum group metals (PGM). Catalytic reactions convert CO, HC and $\hbox {NO}_\textrm{x}$ to $\hbox {CO}_{2}$, $\hbox {N}_{2}$ and $\hbox {H}_{2}\hbox {O}$^8^. The catalytic conversion of pollutants occurs through oxidation-reduction (redox) reactions catalyzed by the active sites on the catalyst surface. These reactions take place at elevated temperatures, typically ranging from $150^{\circ }$C to $300^{\circ }$C. The efficiency of catalytic converters is strongly dependent on their operating temperature. Below a certain temperature threshold known as the light-off temperature, catalytic activity is limited, and emissions reduction is inefficient^9^. During engine startup or when the catalytic converter is cold, exhaust temperatures are low, and the catalyst may not be active. This results in increased emissions of pollutants until the catalytic converter reaches its light-off temperature. Consequently, it is of utmost importance to understand how a given catalytic converter operates within a certain combustion system, to make sure that it reaches its effective temperature range as quickly as possible.

Flow patterns in these catalytic converters are complex due to the space constraints characteristic of automobile applications. More specifically, flow maldistribution is typically unavoidable, for example, when there is a sharp bend in the inlet pipe of the catalytic converter or if the inlet cone angles are very large to save space^10^. The presence of flow maldistribution implies that certain areas at the catalytic converter inlet receive higher velocities, while other areas see lower velocities. Consequently, different regions of the catalyst yield varying rates of species conversion, temperature and ageing. The reactor may not be utilized properly, or it may have to be over-dimensioned to perform its intended function at all operating conditions. Flow maldistribution is therefore, a key phenomenon to understand to accurately predict the long-term behavior of exhaust gas aftertreatment systems.

Physical processes like adsorption, desorption, surface reactions and gas phase reactions, convective and diffusive transport of mass, momentum and heat occur in the catalytic converters^8^, and these transport processes and kinetics span large timescales and are coupled and non-linear. These phenomena can be represented by partial differential equations in multiple spatial coordinates and time. Mathematical modeling and numerical simulations are often used to predict emissions concentrations at the catalyst outlet, by solving conservation equations of mass, momentum, energy, and species^11–15^. With the growing focus on enhancing fuel economy for reducing carbon footprint, while reducing tailpipe emissions of regulated pollutants simultaneously, the ability to perform real-time (or faster) simulations of after-treatment systems is becoming crucial for implementing advanced fueling strategies and optimization algorithms. To this end, simplified models and reduced order models are desired for their speed that make them amenable for usage in real-time monitoring and control. The bottleneck in these models is to include the non-linear coupling between transport phenomena and chemical reactions across multiple spatial and temporal scales with speed and accuracy. The first simplification and a common assumption in the mathematical modeling of catalytic converters is that all the channels of the catalytic converter are exposed to identical inlet conditions and that the monolith is adiabatic. This simplification allows the modeling and analysis of one “*single channel*” of the many hundreds of channels actually present. Such a model is termed a single channel model (SCM). The computational cost required is much lower than a full 3D simulation, as a conventional single channel model is only discretized along the axial direction of the channel and thus constitutes a 1D description of only a part of the complete geometry. Also, the concentrations and temperatures in the bulk and on the channel surface are assumed to be the same (pseudo-homogeneous model)^16^. An important refinement is the 1+1D modeling, where the discretization is made lengthwise and through the radial thickness of the wall^17–19^. Further, heat and mass transport resistances are used to estimate the gradients in the longitudinal and transverse directions^20^. Other developments include incorporating detailed kinetics^21^ and methodology for parameter estimation of kinetic constants^20,22^. Reduced order models have been developed from 1+1D models by computing washcoat diffusional effects, using Jacobian to relate the rate of formation of species and concentrations of the species in the washcoat^23^, and by averaged convection-diffusion-reaction (CDR) equations that reduce the transverse degrees of freedom in the monolith^24^. These reduced order models constitute different versions of a 1D-SCM.

Warm-up of the catalyst influences the catalyst light-off and is strongly dependent on the heat transfer coefficient^25^. Processes like coldstart and flow maldistribution pose challenges to this SCM. Under these conditions, a 3D computational fluid dynamics (CFD) solution is preferred^25,26^. But the 3D nature of the simulation poses challenges in terms of the computational effort, as the simulation time required is significantly longer than the corresponding real time. Several approaches like porous media assumption^27–30^, symmetry in geometry^27^, use of global kinetics for the catalytic reactions^31^, and effectiveness factor models^32^, have been tried by researchers to reduce the computational load. Despite these simplifying assumptions, the computational load prohibits the use of 3D-CFD models for control and monitoring use. It is then desirable to have a model that is as computationally effective as the 1D-SCM, while having the accuracy of the 3D-CFD.

In our earlier paper^31^, we described a methodology of deriving multi-channel models for a reactive simulation combing transient CFD simulations and multi variate data analysis methods like principal component analysis (PCA), D-optimal design and weighted least squares (WLS). The calibration set consisted of transient inlet profiles in mass flowrate, temperature and species concentration. The test cases were either interpolated subsets or time-shifted subsets. The accuracy of the predictions of the multi-channel model were comparable with that of the CFD simulations, and remarkably better than the single channel model. However, the method requires access to the reactive transient CFD data for identifying the channels using PCA and D-optimal design. It is clear that it would be very advantageous if a reduced order model could be trained using only steady-state reactive CFD data, as this would significantly decrease the time needed to acquire the training data.

In this work, we present a novel methodology in which steady-state reactive 3D simulations are combined with nonlinear least-squares optimization in the development of a *pseudochannel model*. The main idea is that flow maldistribution is present even in steady state, thus offering a possibility to avoid computationally costly transient CFD simulations in the correction of a conventional SCM. By characterizing the flow maldistribution in relatively computationally affordable steady state CFD simulations, it becomes possible to devise a pseudo-SCM that does not represent any particular physical channel, but which maps the average inlet conditions to those producing the correct average conversion at the catalyst outlet. The resulting model can then be used in realistic systems with flow maldistribution over a wide range of temperatures.

We first present the basis of the formulation of the nonlinear least squares model and the conservation equations for the CFD modeling, along with the description of the geometry and our test cases. We finally present the performance comparison of the pseudochannel model against the 3D-CFD and 1D-SCM models for various test cases, both at steady state and in transient operation. In this study, ’transient operation’ refers to transitions between different steady states. While it is computationally feasible to simulate a full transient response, such as a complete WHTC (World Harmonized Transient Cycle), such simulations are highly resource-intensive and were beyond the scope of the current study. We present the inferences and limitations of the proposed pseudochannel model, followed by the conclusions and identified developments required for further improving the performance of pseudochannel models.

## Proposed methodology - pseudochannel model

In a chemical reactor, the species conversion is a nonlinear function of the reaction rate and the residence time. Reaction rate is generally expressed in terms of Arrhenius form. This is shown as

1 $$\begin{aligned} \chi _i = f(\tau , k_0 \exp \left( \frac{-E_a}{RT} \right) [C_A]^a) \end{aligned}$$

where, $\chi _i$ is the conversion of species *i* and is defined as $1 - C_{A_i}/C_{A_0}$ (which is the ratio of moles of reactant *A* reacted (mol/m^3^) to that of moles of *A* fed initially (mol/m^3^)), $\tau$ is the residence time (s), $k_0$ is the pre-exponential factor (m^3^,K,mol^-1^,s^-1^), $E_a$ is the activation energy (J/mol), *R* is the universal gas constant (J/mol,K), *T* is temperature (K), and $[C_A]^a$ is the functional form of the species concentration.

In this context, an SCM is an elegant and robust 1D-model that is used to predict the species concentration at the exit of the catalytic converter^33^. In the SCM, it is assumed that every channel experiences the same inlet conditions at any given time. Under adiabatic conditions, the heat of reaction, if exothermic, heats up the gases to increase the temperature. When there is flow maldistribution, some of the channels experience a larger flow, while some other channels experience a smaller flow. This causes differences in channel temperatures and residence times. Hence, the conversion is a (non-linear) function of the flow maldistribution in addition to residence time and temperature as given in Equation 1.

The conversion in a realistic system is the mass weighted average of the spatially distributed concentrations at the reactor outlet. When a 3D-CFD model is used to represent a realistic catalytic converter, it describes the axial and radial flow field and the heat and mass transfer effects in great detail, and for the purpose of the current work, it can thus be considered to provide the true solution to the flow field. Applying an SCM to a realistic system will yield erroneous outlet predictions in the presence of flow maldistribution, owing to the assumption of uniform inlet conditions and the neglect of radial heat transfer. The predicted conversions will then be different from those in the real system or those predicted from 3D models. At constant temperature, a pseudochannel model can be envisaged as an SCM that will give the same conversion as the system with flow maldistribution, at a different (pseudo-physical) residence time. Thus, for realistic systems with flow maldistribution, Equation 1 is recast as

2 $$\begin{aligned} \chi _i = f(\tau , k_0 \exp \left( \frac{-E_a}{RT} \right) [C_A]^a, \text {geometry}). \end{aligned}$$

The objective function required in the derivation of the pseudochannel model will then be formulated as follows: find the residence time of an SCM that will provide the same conversion of the chosen species at steady state reactive conditions at a known inlet temperature.

3 $$\begin{aligned} \tau _{\text {SCM}} = \min \sum _{i=1}^{n_{\text {sp}}} f(\chi _{i_{\text {CFD}}} - \chi _{i_{\text {SCM}}})^2 \end{aligned}$$

where $n_{sp}$ is the species that are considered in the objective function, namely, CO, $\hbox {C}_{3}\hbox {H}_{6}$ and NO.

Here, the true solution (velocity, temperature and species concentration) for the flow field is obtained from steady-state reactive 3D-CFD simulations. The unknown variable (the residence time for an equivalent 1D-SCM) can be obtained by non-linear optimization, by minimizing the error between simulated data from the detailed 3D-CFD model and the simplified lumped model (1D-SCM).

As the conversion is a non-linear function of residence time and temperatures, the Levenberg-Marquardt algorithm can be used to obtain the solution of Equation 3.^34,35^ This algorithm combines the advantages of the Gauss-Newton algorithm and the gradient descent algorithm, by interpolating between these two approaches, making it particularly effective for minimizing the sum of squared differences between observed and predicted values.

In the formulation of the pseudochannel model, the objective function is given by Equation 3 and is solved using the lsqnonlin function in MATLAB^36^. This method needs an appropriate initial guess and bounds are not needed to be specified. The mixed cup values at the inlet of the catalyst from the CFD results can be used as the initial guess.

This method involves a test matrix that is populated with conversions of species obtained from steady state simulations specifying gas hourly space velocities (GHSV), sweeping across a range of temperatures covering light-off conditions of species and extending to higher temperatures, where species are fully converted.

## Results

Steady state reactive 3D-CFD simulations were performed, with five reactions involving nine species. The test matrix consisted of four gas hourly space velocities (GHSV) and ten temperatures spanning from 350 K to 575 K, chosen to span a relevant range of operating conditions^28^. These temperatures include light-off temperatures for the species CO, $\hbox {C}_3\hbox {H}_{6}$ and NO and temperatures that are attained when the catalyst is sufficiently heated. This matrix serves as the “calibration” case to generate the velocity map that has to be specified at the inlet for a pseudochannel. Two other sets of transient simulations, in terms of step changes in mass flowrate and temperature, form additional test cases to further study the performance of the pseudochannel model.

### Steady state simulations

**Fig. 1:**
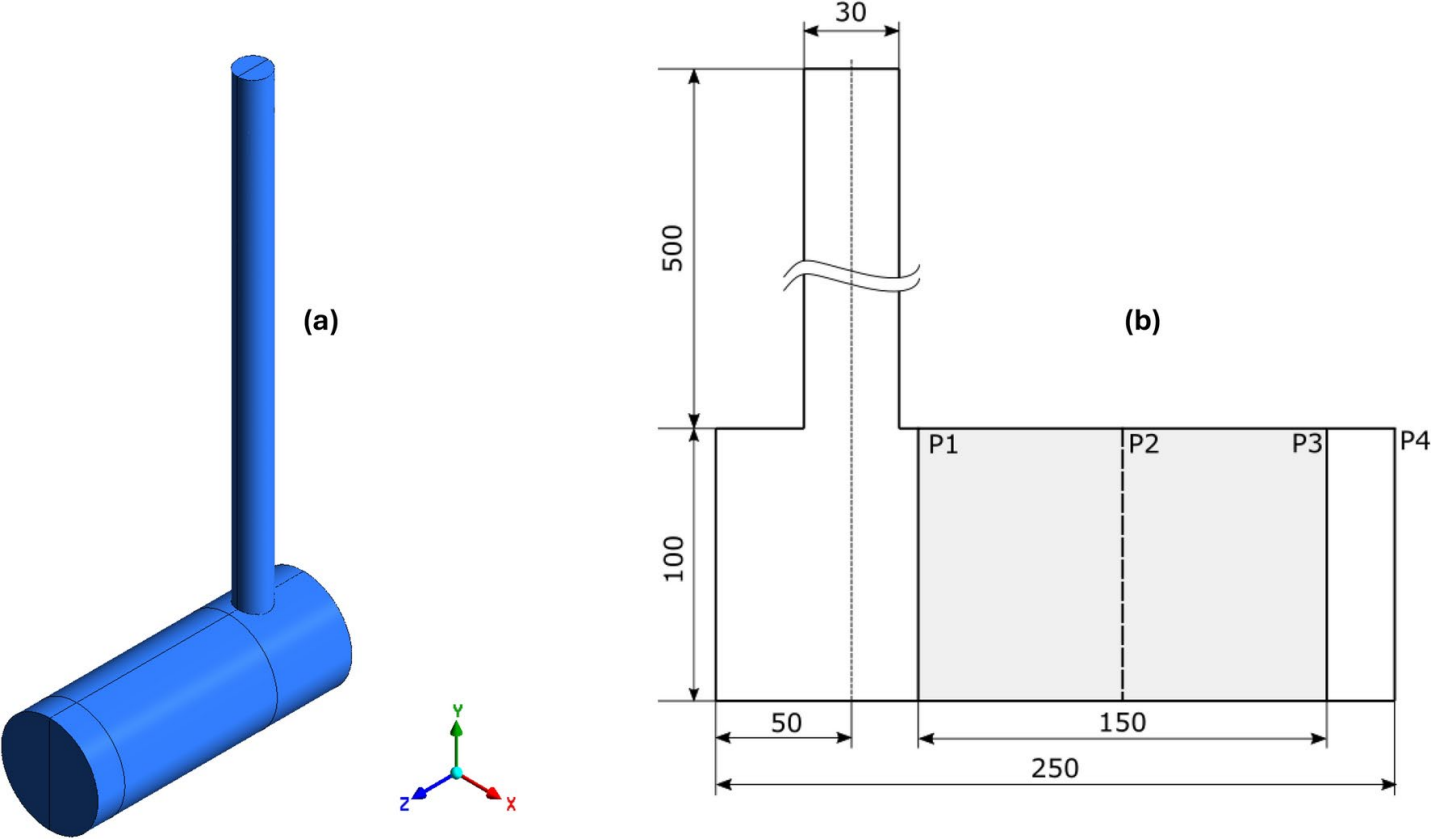
Representation of the geometry. (**a**) is the 3D representation of the geometry. (**b**) is the representation of planes in the geometry. Three distinct regions can be identified in the geometry: a vertical section and unshaded horizontal section and shaded horizontal section. Catalyst is specified as the shaded region. The flow starts at the vertical most point in the geometry, then descends down. Before exiting at P4, the flow experiences a bend prior to plane P1. A dead volume is also present at the left end of horizontal section.

CFD solution variables were collected at the inlet plane P1 and at the outlet plane of the catalyst P3 as shown in Figure 1.

**Fig. 2:**
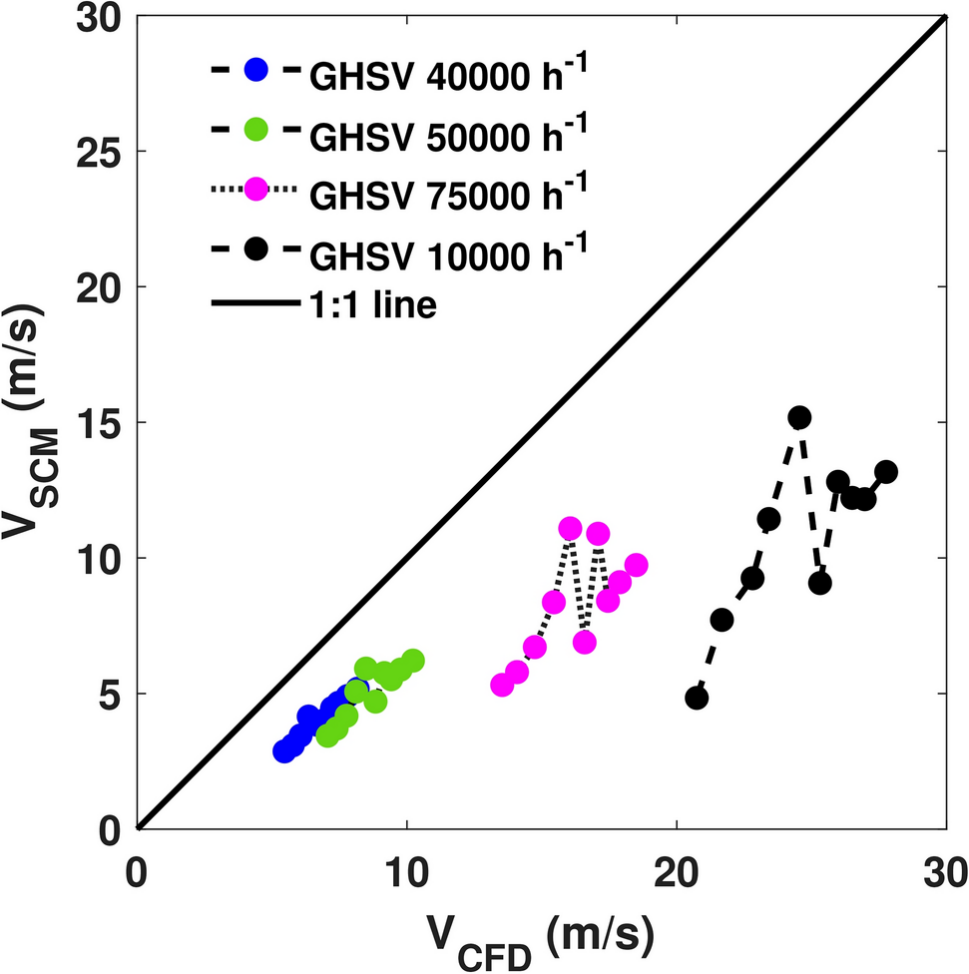
Mapping of velocity of the pseudochannel with the corresponding mixed-cup inlet velocity from 3D-CFD simulations for ten different inlet temperatures (350K to 575K in steps of 25K) at four different GHSVs. The 1:1 line is the diagonal line that specifies equal residence times of SCM and CFD. As the mapped points lie below this 1:1 line, the residence time in the pseudochannel will be longer, requiring the species to reside in the reactor longer to attain the same conversion as in 3D-CFD.

The value of mixed cup velocity at the inlet plane P1 is used as the initial guess in the nonlinear least squares optimization. The desired conversions of the three species were obtained from the outlet plane P3 of the catalyst. lsqnonlin was run for all the 40 operating points and the corresponding velocity of the pseudochannel is plotted against the mixed cup inlet velocity as shown in Figure 2. Here, it should be stressed that the mixed cup velocity is the one that would have been used in a conventional SCM, whereas the pseudochannel velocity is the velocity to use with an SCM to obtain the best possible prediction when assessed against the full-scale CFD simulation.

Thus, Figure 2 shows the mapping of velocities of the SCM model with a corresponding 3D-CFD model at steady state. At lower GHSV, the behaviour is linear and becomes slightly non-linear, due to effects of flow-maldistribution. In our earlier paper^28^, we had noticed that the lower sections of the geometry experience higher flows due to the jet ensuing from the inlet section, before undergoing a sharp bend before the inlet plane P1. This effect becomes more pronounced at higher temperatures, due to the interaction of flow and thermal inertia. From Figure 2, we can see that the pseudochannel is correlated with the inlet mixed cup velocity of the 3D-CFD. The values of this mapping can be used in forms of a look-up table or in functional form (piece-wise linear mappings). It can be seen that the pseudochannel needs to have a lower velocity for increased residence time to achieve conversion predictions similar to the 3D-CFD values. With different initial guesses, the model converged to the same end result, assuring unique solution for a set of temperature and conversion values in the objective function.

**Fig. 3:**
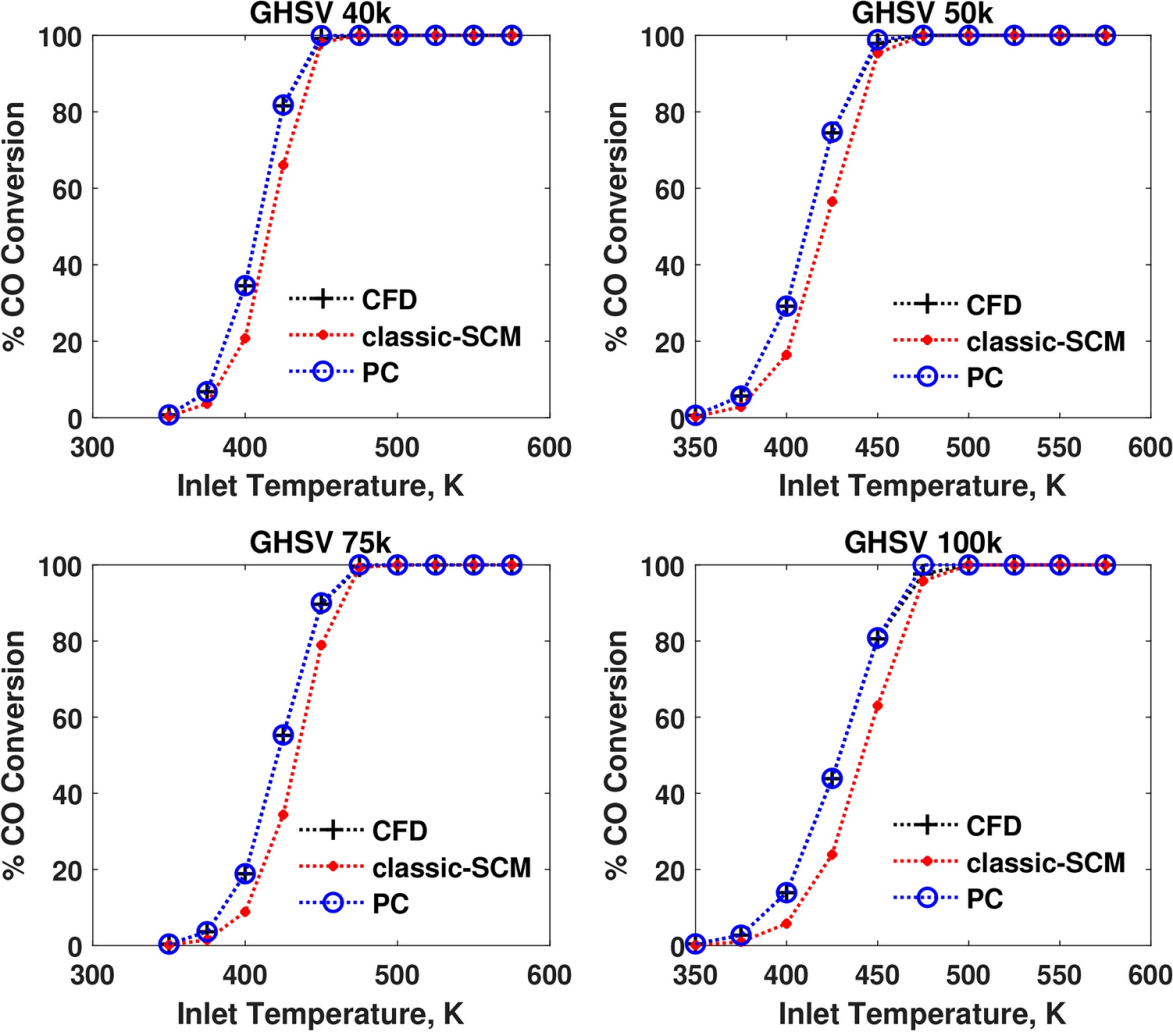
Comparison of performances in terms of CO conversion at four different GHSVs.

**Fig. 4:**
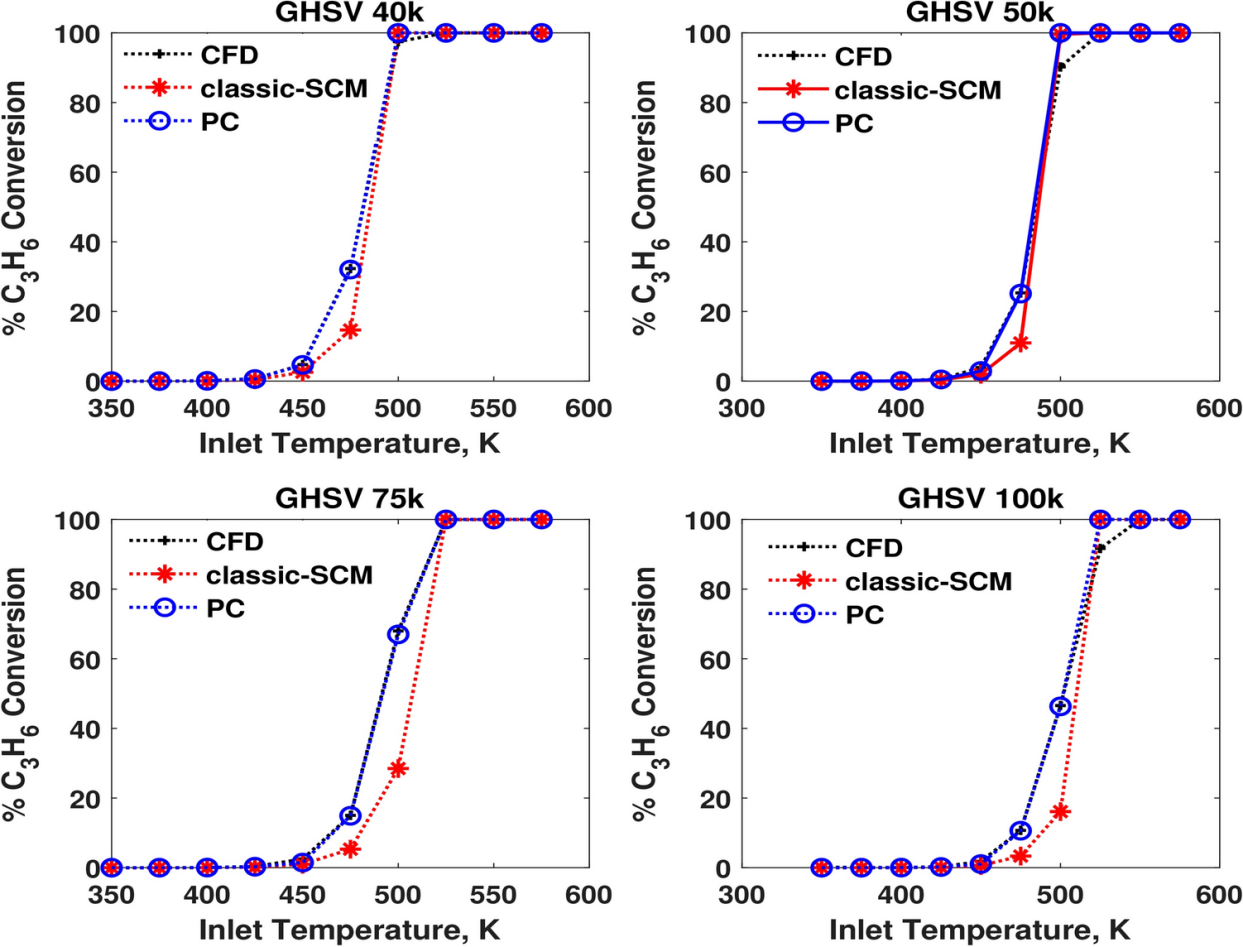
Comparison of performances in terms of $\hbox {C}_3\hbox {H}_{6}$ conversion at four different GHSVs.

**Fig. 5:**
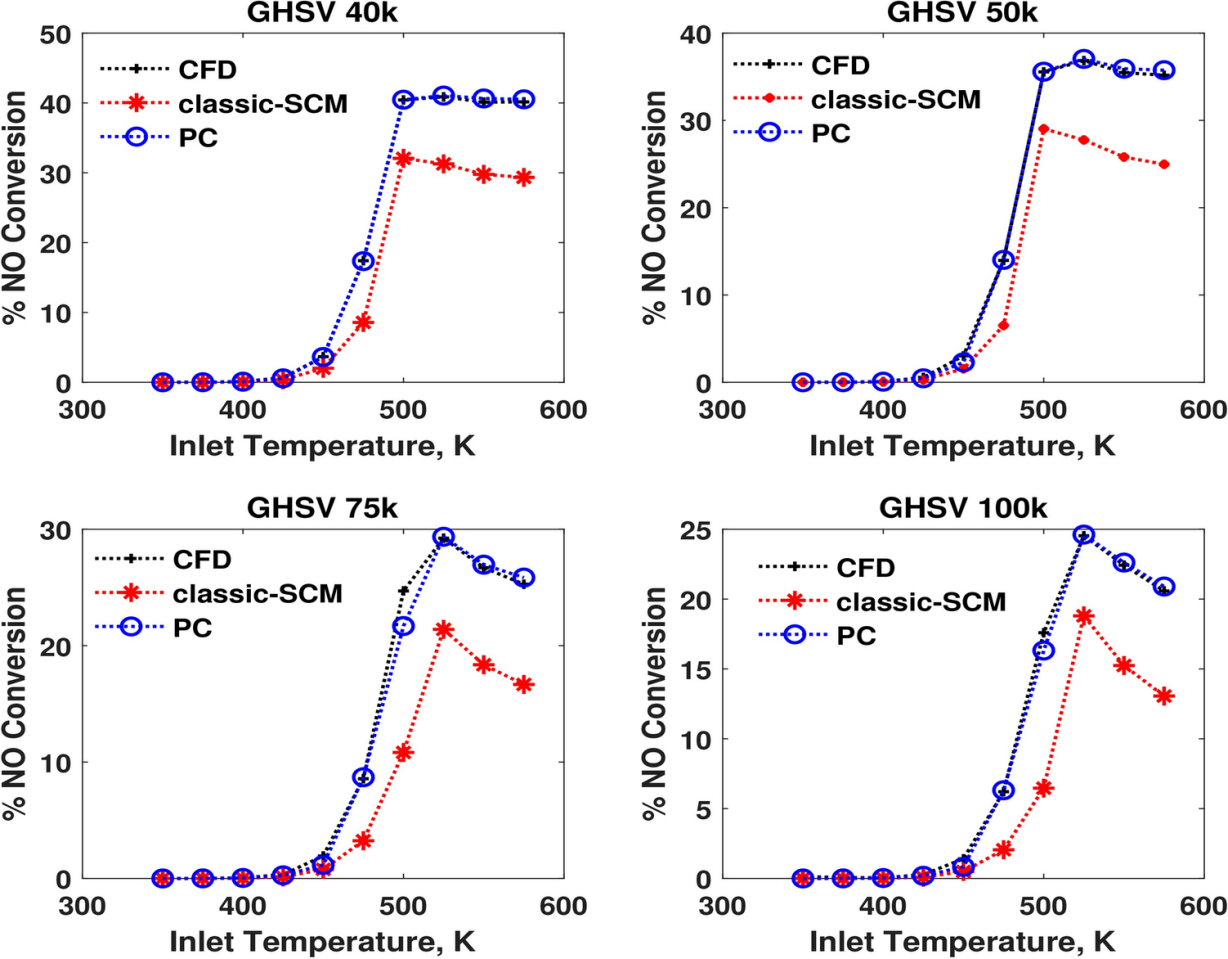
Comparison of performances in terms of NO conversion at four different GHSVs.

The performance of the pseudochannel under steady state conditions is compared with that of the 3D-CFD and a conventional 1D-SCM in terms of outlet temperature and species conversion in Figures 3,  4, and 5. It is surprising how well a one-parameter model seems to be able to explain the non-linear flow maldistribution effects, predicting conversions more similar to that of a 3D-CFD simulation.

Figures 3,  4, and  5 show that the conversions of the pseudochannel match more closely with the CFD values and are better than the SCM results. The light-off trends are also captured accurately. As temperature varies from 375 K to 575 K, the species light-off of CO, followed by $\hbox {C}_3\hbox {H}_{6}$, occur and NO has a maximum conversion at intermediate temperature and the reversible oxidation to $\hbox {NO}_{2}$. As CO reaches light-off condition in terms of temperature, the conversion of CO increases rapidly with temperature. This increase of the reaction rate, in turn, increases the temperature of the catalyst itself via the heat of reaction. Once CO conversion becomes saturated, the contribution of CO to the gradients of the objective function decreases. When CO is completely converted, it does not contribute to the gradients of the objective function. This trend repeats for other species like $\hbox {C}_3\hbox {H}_{6}$ and NO. The fitting method is still efficient, such that even when the species conversion progressively reaches saturation, the objective function is robust in predicting the residence time for pseudochannel. The temperature sweep at different values of GHSV provides the mapping, predicting the values and trends of conversion and light-off temperatures under the steady state conditions.

### Transient simulations

Having tested the effectiveness of the pseudochannel model, the next test is to see the performance of the model when the system moves from one steady state to another, in terms of velocity (acceleration) and temperature (light-off). In general, flow maldistribution becomes more pronounced at higher mass-flow rates.

**Fig. 6:**
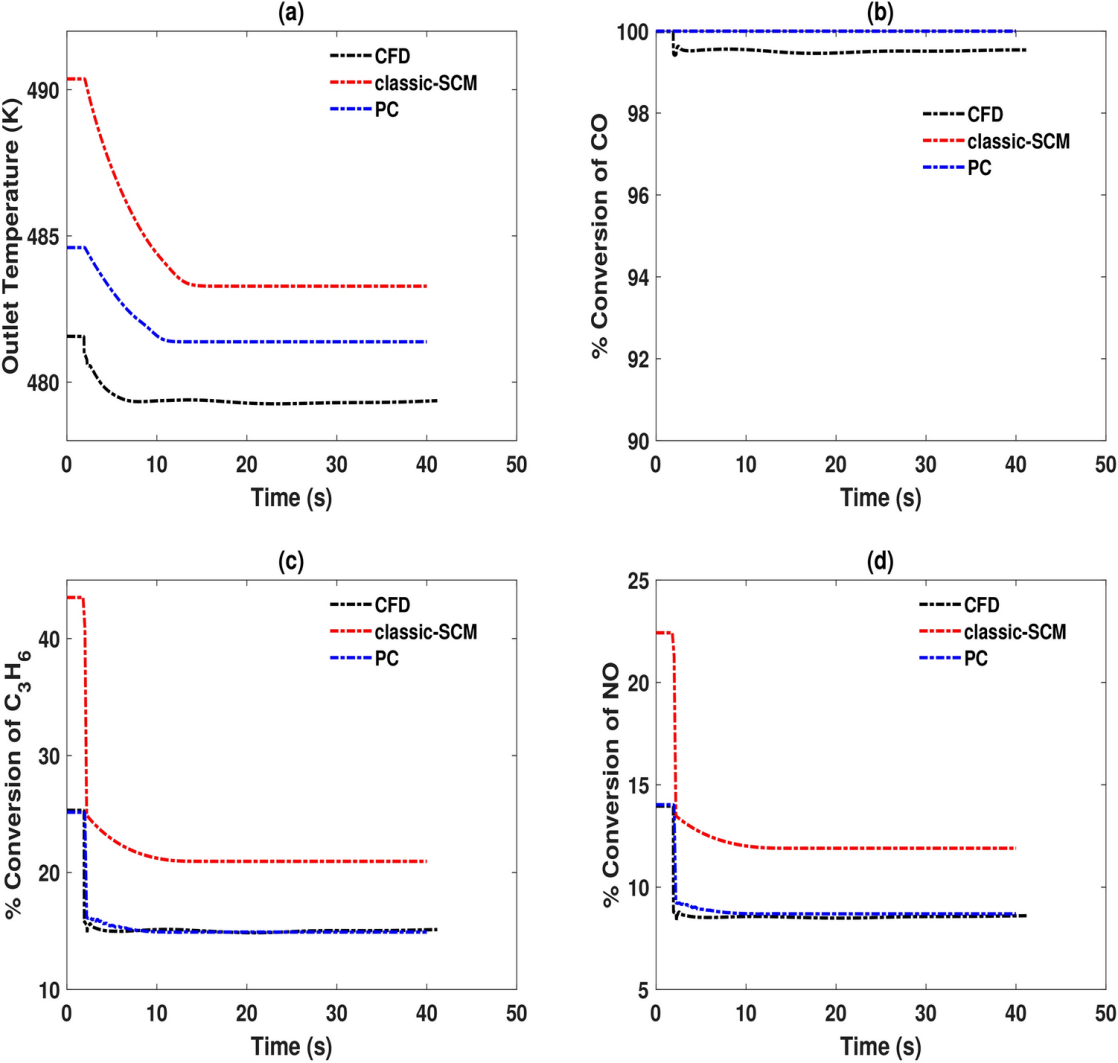
Comparison of performances of 3D-CFD (CFD), 1D-SCM (classic-SCM) and pseudochannel (PC) models in terms of outlet temperature and species conversions for a transient velocity step at the inlet. The step change in mass flow rate occurs at $t = 2$ s, from 0.018 kg/s to 0.028 kg/s, while holding temperature constant at 475K. This temperature is higher than the light-off temperature of CO. Panel (**a**) presents outlet temperature, panels (**b**), (c) and (**d**) are the conversions of species CO, $\hbox {C}_3\hbox {H}_{6}$ and NO respectively.

Figure 6 shows the outlet temperatures and species conversions of CO, $\hbox {C}_3\hbox {H}_{6}$ and NO when a step change from 0.018 kg/s to 0.028 kg/s is made at $t = 2$ s. Qualitatively, the prediction trends are similar for all the models, for temperature and species conversion. Panel (a) shows a drop in temperature after the transient step change in mass flowrate. With an increase in the mass flowrate, the residence time decreases. As a consequence, the rates of reaction decrease, leading to a decrease in the outlet temperature from smaller exotherms. This trend is seen with all the three models. The temperature and residence time effects also influence species conversions. The predictions of the pseudochannel model are closer to the CFD predictions than 1D-SCM except for CO conversion. Since the inlet temperature is 475K, the conversion of CO is very high, nearly complete. 3D-CFD profile of CO conversion is lower than the 1D predictions due to that the flow maldistribution changes with the incoming mass flow rate. The temperature distribution is also modeled via radial heat transfer in the monolith in 3D-CFD, unlike the 1D models. With the mapped velocity applied to the pseudochannel, the predictions of this model are closer to the 3D-CFD results than the conventional SCM approach both before and after the step.

An important aspect of the $\hbox {NO}_\textrm{x}$ conversion is its dependence on complex reaction pathways and sensitivity to operating conditions. We can see from the kinetics that NO participates in the reduction reaction with $\hbox {C}_3\hbox {H}_{6}$ and in reversible oxidation to $\hbox {NO}_{2}$. According to the Le Chatelier-Braun principle, any disturbance in conditions such as temperature, flowrate, or concentration will shift the equilibrium of the reversible $\hbox {NO} +0.5 \hbox {O}_2 \longleftrightarrow \hbox {NO}_{2}$ reaction to counteract the disturbance. This interplay can make the concentration of NO highly sensitive to operating conditions. The simultaneous occurrence of reduction and reversible oxidation reactions means NO participates in competing pathways. Further, the conversion of NO is influenced by reaction kinetics and transport effects, and stoichiometric limitations (availability of $\hbox {C}_3\hbox {H}_{6}$ and NO) can affect the observed NO levels. Despite these challenges, the developed pseudochannel model predicts conversions in good agreement with 3D-CFD simulation results.

**Fig. 7:**
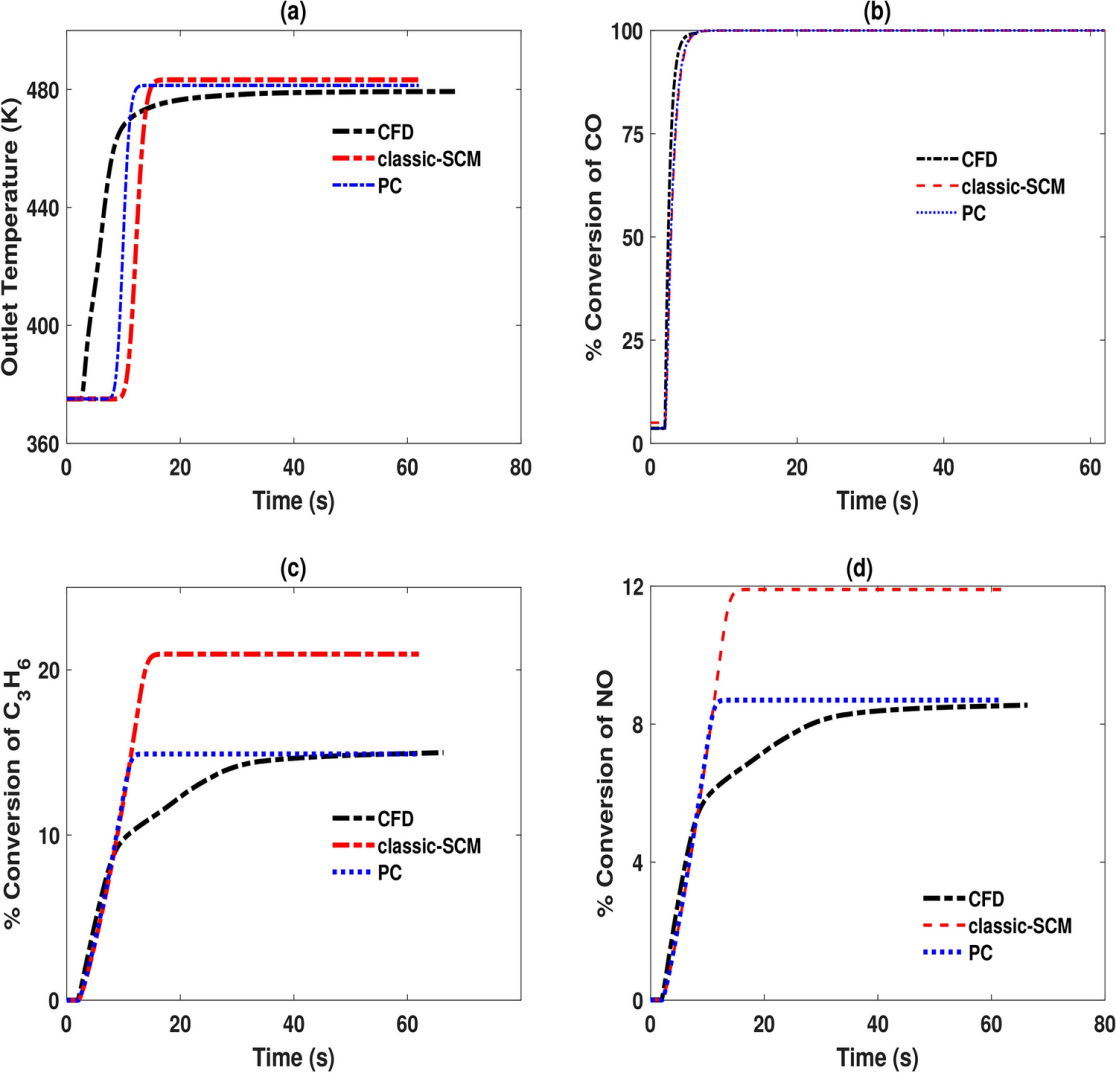
Comparison of performances of 3D-CFD (CFD), 1D-SCM (classic-SCM) and pseudochannel (PC) models in terms of outlet temperature and species conversions for a transient temperature step at the inlet. The step change in temperature occurs at $t = 2$ s, from 375K to 475K, while holding mass flow rate constant at 0.028 kg/s (corresponding to a GHSV of 50000 $\textrm{h}^{-1}$). This temperature step covers temperatures before and after the light-off temperature of CO. Panel (**a**) presents outlet temperature, panels (**b**), (**c**) and (**d**) are the conversions of species CO, $\hbox {C}_3\hbox {H}_{6}$ and NO respectively.

Figure 7 shows the results of the temperature step transient. The step change occurs at $t = 2$ s, where the temperature at the inlet increases from 375K to 475K. The pseudochannel predictions agree well with 3D-CFD predictions both before and after the step and outperforms the conventional SCM for the temperature transients too. An interesting feature is the thermal lag behaviour. The dynamic response observed in the 3D-CFD is a natural outcome of the model, as the radial heat conduction responsible for the emergence of “history effects” is accounted for. The SCM variants are characterized by a lumped thermal mass and therefore cannot predict the exact dynamics of the transient response to a change in temperature. This implies that as the inlet temperature changes, the CFD model is sensitive enough to predict the variation and retention times that results. The change is both felt sooner than the SCM variants (for the channels receiving the highest inlet velocities) and lasts for longer (for the channels receiving the lowest velocities). Although the pseudochannel SCM can produce predictions of conversion that are better matches to the CFD at the steady states before and after the temperature step, it cannot accurately describe the transient response of the reactor to these types of inlet variations.

## Computational efficiency

All simulations, both 1D-SCM and 3D-CFD were carried out on an i7-processor powered desktop computer having 64 GB RAM. Reactive steady state simulations were carried out progressively starting from low temperature (350K) to high temperature (575K). The initialization was done at 350K and the converged solutions were subsequently used for the next higher temperature setting. The first set of steady state solutions were obtained in 18 minutes, thereafter, this solution time for other temperatures of the order of 30 minutes (the reason that higher temperatures require longer simulation times is due to the stiff kinetics). The 1D-SCM simulations were carried out with the ode15s solver in MATLAB. Nonlinear least squares optimization was also performed using the lsqnonlin function in MATLAB. The simulation time for the 1D-SCM cases was of the order of 2 minutes. The mapping procedure using nonlinear least squares was completed in 2-5 minutes for low temperatures (temperatures less than 450K) and high temperatures respectively (temperatures from 450K). The total computational effort is detailed in Table 1. It is clear that the pseudochannel SCM has the same computational cost as a conventional SCM, but delivers results that are much more similar to full 3D-CFD. The only additional computational cost for deriving the velocity mapping underpinning the pseudochannel SCM comes from the steady-state CFD cases (30 minutes) and the fitting procedure (5 minutes). The mapping is geometry-specific, and it need only be derived once per geometry (and kinetic mechanism). From Table 1, the ratio of computational times for 3D-CFD and the pseudochannel model is 100 : 1, which indicates that CFD-quality predictions of catalyst behavior in realistic flow maldistribution cases can be obtained at SCM-cost. These results open up for several applications like virtual calibration platforms, monitoring and control^37^, which today still rely on models of limited accuracy to achieve sufficient computational efficiency.

**Table 1:** Comparative computational costs for steady-state and transient simulations and other associated processing steps.

Details	3D-CFD	1D-SCM	Pseudochannel
Steady-state reactive simulation	30 minutes	2 minutes	2 minutes
white lsqnonlin optimization	-	5 minutes	-
Transient reactive simulations	3 hours	5 minutes	5 minutes

## Discussion

In the view of stringent emission legislations, control algorithms dependent on the availability of ever faster and more accurate models. Traditionally, 1D-SCM are being used to get the exit concentrations of the emissions. These models are robust and offer good predictions when the conditions at the inlet of the catalyst are uniform. Flow maldistribution and coldstart are conditions which require models that are more detailed than the 1D-SCM. CFD models can be used in these conditions to provide accurate results, but their use in control and monitoring is limited due to the large computational effort. Reduced order models provide the required accuracy at lower computational cost. In this work, a pseudochannel model is developed from steady reactive simulations through mapping of residence time and kinetics from 3D-CFD onto 1D-SCM. A one-parameter model in terms of residence time is capable of predicting conversions of the species by accurately characterizing the flow maldistribution via an alteration of the inlet boundary condition to a conventional SCM. The pseudochannel does not represent any physical channel in the monolith, but is a fictitious channel whose inlet conditions have been arrived at via an optimization procedure that determines the inlet velocities an SCM must have to provide the equivalent exit conversion as a full-scale 3D-CFD simulation.

In place of the herein proposed one-parameter mapping, it is also possible to devise a two-parameter mapping by varying temperature and residence times simultaneously. This will produce a fictitious temperature and velocity set that has to be specified for the pseudochannel. As the thermal mass is significantly larger than the flow inertia, the large time constants of the thermal inertia bring about slower responses in the temperature field than in the velocity field^28^. Smaller velocity time constants make the system more sensitive to changes in inlet velocity. Moreover, this two-parameter mapping introduces additional constraints to be satisfied increasing the complexity. This can be tested in real driving cycles such as the World Harmonic Transient Cycle (WHTC) or Worldwide Harmonised Light Vehicle Test Procedure (WLTP). The former is a technical regulation under more stringent emission regulations like Euro VI, and the latter is a set of standardised laboratory test procedures.

The intrinsic ability to incorporate thermal history effects renders the CFD model superior to the 1D-SCM model variants that do not account for radial heat transfer. Consequently, the 1D-models show delayed responses to the changes in inlet temperature. To remedy this deficit in 1D-SCMs, there would be a need to develop model frameworks that represent the distribution of retention times of the real-world system, along with the intercommunication between different parts of the reactor through the solids material.

In terms of computational time, the pseudochannel model is as efficient as the 1D-SCM and much better than the 3D-CFD, while still producing results as accurate as the 3D-CFD. Being computationally efficient, pseudochannel models can possibly be further optimized for realtime monitoring and control. The pseudochannel model can be used for practical applications despite the limitations of thermal lag, as the velocity transients are captured well and velocity transients have smaller time constants.

## Conclusion

A monolithic reactor has hundreds of parallel channels, and the resolution of all these channels in a simulation model is computationally very costly. This situation restricts the use of full-scale models in real-time monitoring and control applications. At the same time, the accuracy offered by such models is superior to that of conventional single-channel models (SCM) that cannot account for flow maldistribution.

In this work, a novel, computationally inexpensive pseudochannel model is presented for emission aftertreatment systems with flow maldistribution. This pseudochannel model is a fictitious single-channel model that more accurately represents the operation of a realistic catalytic converter, as compared to the conventional SCM. The key component of the pseudochannel model is a map of residence times that compensate for flow maldistribution effects. This map is obtained from an optimization problem in which the pseudochannel model is trained to reproduce species conversions in steady-state CFD simulations at different flow rates, holding temperature constant.

The performance of the developed pseudochannel model is compared with the full 3D-CFD solutions and a conventional SCM for the same nominal inlet velocities. In all the cases, the performance of the pseudochannel model is significantly better than the conventional SCM model. Relatively cheap steady-state reactive 3D-CFD simulations are thus used to derive a computationally effective pseudochannel model, offering a computationally inexpensive methodology for realistic systems with flow maldistribution. It is confirmed that the pseudochannel model agrees very closely with the 3D-CFD results under steady-state conditions, as well as for a velocity transient case. However, the sensitivity to capturing variation in retention times in a temperature step is limited due to the lumping of the thermal mass and the absence of radial heat transfer modelling. This is a known deficit in the conventional SCM and it is not rectified by the pseudochannel approach. It is suggested that this work be expanded to include thorough testing of the proposed procedure using a realistic driving cycle, such as the WHTC, and compare the performance of this model with real-time exhaust data and to explore the effectiveness of using smaller calibration data sets, and possible implementation of control algorithms for processes like $\hbox {NH}_{3}$-dosing in SCR.

## Methods

We use steady state reactive CFD simulations in combination with non-linear least squares optimization to develop the pseudochannel model. An oxidation kinetic scheme in a catalytic converter is chosen and is represented by global kinetic rate equations from Pandya *et al.*^38^. It consists of five reactions occurring in the diesel oxidation catalyst, namely the oxidation of CO, $\hbox {H}_{2}$, $\hbox {C}_3\hbox {H}_{6}$, reversible oxidation of NO to $\hbox {NO}_{2}$ and reduction of NO by $\hbox {C}_3\hbox {H}_{6}$ as shown in Table 2. An exhaust gas mixture consisting of nine gases (CO, $\hbox {O}_{2}$, $\hbox {C}_3\hbox {H}_{6}$, NO, $\hbox {NO}_{2}$, $\hbox {CO}_{2}$, $\hbox {H}_{2}$, $\hbox {H}_2\hbox {O}$ and $\hbox {N}_{2}$) is used in the reactive simulations. The ideal gas law is used for all gases, and all thermo-physical properties are treated as functions of temperature. To this end, we follow the steps in a generic CFD simulation. These are described in the subsections below. The performance of the pseudochannel model is compared with the full CFD model and a 1D-SCM model. The methodology is described here with the model equations.

**Table 2:** DOC kinetics and global rate expressions by Pandya et al.^38^. $X_i$ represents the mole fraction of species *i*, $k_j$ are rate coefficients, $K_j$ are adsorption equilibrium constants, and $G_j$ are inhibition terms. *R* is the universal gas constant, and *T* is the temperature (K).

Reaction	Global rate expression (kmol/m_cat_^2^, s)
R1: $\hbox {CO} +\frac{1}{2}\hbox {O}_2 \longrightarrow \hbox { CO}_{2}$	$\dfrac{k_1 X_{\hbox {CO}} X_{\hbox {O}_{2}}}{G}$
R2: $\hbox {C}_3\hbox {H}_{6} +\frac{7}{2}\hbox {O}_{2} + \hbox {2NO} \longrightarrow \hbox {3CO}_2 + \hbox {3H}_2\hbox {O} + \hbox {N}_{2}$	$\dfrac{k_2 X_{\hbox {C}_3\hbox {H}_{6}} X_{\hbox {NO}} X_{\hbox {O}_{2}}}{G G_4}$
R3: $\hbox {C}_3\hbox {H}_{6} +\frac{9}{2}\hbox {O}_2 \longrightarrow \hbox {3CO}_2 + 3 \hbox {H}_2\hbox {O}$	$\dfrac{k_2 X_{\hbox {C}_3\hbox {H}_{6}} X_{\hbox {O}_{2}}}{G}$
R4: $\hbox {NO} +\frac{1}{2}\hbox {O}_2 \longleftrightarrow \hbox {NO}_{2}$	$\dfrac{k_3\left( X_{\hbox {NO}} X_{\hbox {O}_{2}}^{0.5} - P^{-0.5}X_{\hbox {NO}_{2}} / K_{EQ}\right) }{G}$
R5: $\hbox {H}_2 +\frac{1}{2}\hbox {O}_2 \longrightarrow \hbox { H}_2\hbox {O}$	$\dfrac{k_1 X_{\hbox {H}_{2}} X_{\hbox {O}_{2}}}{G}$
**Adsorption inhibition factors:**	
$$G = G_1 G_2 G_3$$	
$$G_1 = \left( 1+ K_1 X_{\hbox {CO}} + K_2 X_{\hbox {C}_3\hbox {H}_{6}}\right) ^2$$	
$$G_2 = 1 + K_3 \left( X_{\hbox {CO}} X_{\hbox {C}_3\hbox {H}_{6}}\right) ^2$$	
$$G_3 = 1 + K_4 X_{\hbox {NO}}$$	
$$G_4 = 1 + K_5 X_{\hbox {O}_{2}}$$	
**Kinetic Constants:**	
$$k_1 = 1.93 \times 10^{11} \exp \left( \frac{-51873}{R} \left[ \frac{1}{T}-\frac{1}{450}\right] \right)$$	
$$k_2 = 1 \times 10^{9} \exp \left( \frac{-90000}{R} \left[ \frac{1}{T}-\frac{1}{450}\right] \right)$$	
$$k_3 = 2.83 \times 10^{7} \exp \left( \frac{-21341}{R} \left[ \frac{1}{T}-\frac{1}{450}\right] \right)$$	
$$K_1 = 648.6 \exp \left( \frac{6574}{R} \left[ \frac{1}{T}-\frac{1}{450}\right] \right)$$	
$$K_2 = 2.21 \times 10^{4} \exp \left( \frac{13226}{R} \left[ \frac{1}{T}-\frac{1}{450}\right] \right)$$	
$$K_3 = 5.792 \times 10^{13} \exp \left( \frac{40000}{R} \left[ \frac{1}{T}-\frac{1}{450}\right] \right)$$	
$$K_4 = 3.63 \times 10^{4} \exp \left( \frac{4482}{R} \left[ \frac{1}{T}-\frac{1}{450}\right] \right)$$	
$$K_5 = 3.679 \exp \left( \frac{-67207}{R} \left[ \frac{1}{T}-\frac{1}{450}\right] \right)$$	
$$\ln (K_{EQ}) = 5.0462 + \frac{6343.4}{T} - 2.2973 \ln (T) + 3.0315 \times 10^{-3} T - 8.2812 \times 10^{-7} T^2 + 1.1412 \times 10^{-10} T^3$$	

3D-CFD simulations were performed in ANSYS Fluent 2022R2. A pressure-based solver is employed to solve the conservation equations for mass, momentum, turbulent quantities, energy, and species. Accurately predicting recirculation patterns in the inlet diffuser, which lead to flow maldistribution, requires a reliable turbulence model. In this study, the SST $\kappa - \omega$ model, which combines the standard $\kappa - \omega$ and $\kappa - \varepsilon$ models, is capable of accurately capturing turbulence across a wide range of Reynolds numbers. This turbulence model uses a blending function to transition from the standard $\kappa - \omega$ model near the wall to the high-Reynolds-number $\kappa - \varepsilon$ model in the outer boundary layer. It is used with the low-Reynolds-number correction and production-limiter options enabled, as this implementation offers improved accuracy over the commonly used $\kappa - \varepsilon$ model.

The low-Reynolds-number correction in the SST $\kappa - \omega$ model is crucial for accurately modeling turbulence near the wall, where the velocity gradients are steep, by accounting for the effects of the viscous sublayer. This correction thus helps to provide an improved description of the flow behavior in the near-wall regions, at the expense of a finer mesh resolution.

### Geometry and mesh

The geometry used in this study is chosen to replicate the flow phenomena in real world exhaust aftertreatment systems (EATS) with bends, recirculation zones, and dead volumes. As the current work is intended as a proof-of-concept of the pseudochannel approach, the main criterion to be met by the geometry is that it should exhibit *sufficient* maldistribution, so as to not underestimate the effects. In other configurations, different flow maldistribution characteristics may be observed in practice^39,40^. The mapping produced (Figure 2) is thus geometry-dependent.

**Fig. 8:**
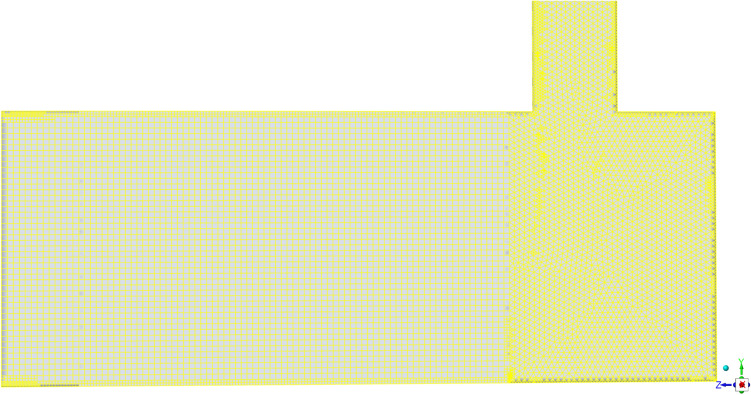
Cut section along the mid-plane of the computational domain, illustrating the mesh resolution inside and upstream the catalyst region. The flow enters from the vertical duct and exits towards the left.

The geometry employed in this study (cf. Figure 1) comprises two sections of uniform cross-section: a vertical section and a horizontal section. The vertical section is a pipe, measuring 60 cm in length and 3 cm in diameter, and it is connected to the horizontal section, which is 25 cm long and 10 cm in diameter. Flow from the vertical section enters the horizontal section at a 90-degree angle. Additionally, the horizontal section contains a catalytic unit consisting of a diesel oxidation catalyst (DOC) with dimensions of 9.5 cm in diameter and 9 cm in length. The DOC features a cell density of 400 cells per square inch (cpsi) and contains precious metal loaded at 10 g/ft^3^, with a washcoat loading of 2.6 g/inch^3^. This geometry has also been used in our earlier work^28^. The flow from the vertical inlet section produces a flow maldistribution at the entry section of the EATS. The full structure when meshed gives nearly seven hundred thousand hexahedral cells with 1.7 million faces and four hundred thousand nodes. The cell volumes ranged from $\mathcal {O}(10^{-13})$ and $\mathcal {O}(10^{-8})$ $\textrm{m}^3$ representing the minimum and maximum volumes respectively. To accurately capture critical phenomena, targeted mesh refinement was employed in key regions, including the near-wall area, interfaces between catalytic and non-catalytic surfaces, and transitional zones such as the junction of vertical and horizontal sections. A cut section of the meshed view is shown in Figure 8. In the catalyst region, the aspect ratio of the cells varied between 0.6 and 2.86, ensuring a balance between accuracy and computational efficiency. A plane of symmetry halves the computational load.

### 1D modeling

The monolith has several hundreds of channels. The channels are the locations where catalytic reactions convert the emission gases to products. 1D modeling is used to obtain the concentration of species and temperature at the exit of the catalyst, by solving species conservation equations and an energy balance equation. It is assumed that the entire monolith can be characterized by a single channel, which in turn is described using the tanks-in-series model.^41^

Component Material Balance for Species *i*

4 $$\begin{aligned} \dfrac{dC_i}{dt} = \dfrac{C_{i0}}{\tau }- \dfrac{C_i}{\tau } + \sum _{j = 1}^{n_{rxn}} \nu _{ij} R_j \end{aligned}$$

Energy Equation

5 $$\begin{aligned} V \rho _{avg} c_p \dfrac{dT}{dt} = q \rho _{avg} c_p(T_0-T) + V \sum _{j = 1}^{n_{rxn}} \left[ (-\Delta H_r^T) \nu _{ij} R_j \right] \end{aligned}$$

Here, $n_{rxn}$ is the total number of reactions, *V* is the volume of a tank in the tanks-in-series model (m^3^), $C_{i_{0}}$ is the concentration ($\mathrm{kmol/m}^3$) of the species *i* in the upstream tank (or at the inlet for the first tank), $C_i$ is the concentration (kmol/m^3^) of the species *i* in the current tank, $T_0$ and *T* are the temperatures (K) in the upstream and current tanks respectively, $\nu _{ij}$ are the stoichiometric coefficients for species *i* in reaction *j*, $R_j$ is the rate of formation or depletion of species in the *j*th reaction (kmol/m^3^,s), $\rho$ is the density of the mixture (kg/m^3^), $c_p$ is the specific heat capacity of the mixture (J/kg, K), and *q* is the volumetric flowrate of the mixture (m^3^/s).

### CFD modeling

The solution of the flow variables like velocities, temperature and species concentrations can be obtained by solving the conservation equations of the Reynolds-averaged formulation of the flow. The URANS (Unsteady Reynolds-averaged Navier-Stokes) equations for continuity, momentum, energy, and species transport can be expressed as follows:

Continuity equation

6 $$\begin{aligned} \frac{\partial \rho }{\partial t} + \frac{\partial \rho \langle u_i \rangle }{\partial x_i} = 0 \end{aligned}$$

where $\rho$ is the fluid density (kg/m^3^), and $u_i$ is the velocity (m/s) in coordinate direction $x_i$.

Momentum balance

7 $$\begin{aligned} \frac{\partial }{\partial t} \left( \rho \langle u_i \rangle \right) + \frac{\partial }{\partial x_j} \left( \rho \langle u_i \rangle \langle u_j \rangle \right) = - \frac{\partial \langle p \rangle }{\partial x_i} + \frac{\partial }{\partial x_j} \left[ \mu \left( \frac{\partial \langle u_i \rangle }{\partial x_j} + \frac{\partial \langle u_j \rangle }{\partial x_i} \right) \right] + \frac{\partial }{\partial x_j} \left( -\rho \langle u_i^{\prime } u_j^{\prime } \rangle \right) \end{aligned}$$

where $\mu$ is the fluid viscosity (Pa,s), *p* is the pressure (Pa), $u_i^{\prime }$ is the velocity fluctuation (m/s) in coordinate direction $x_i$, and $-\rho \langle u_i^{\prime } u_j^{\prime } \rangle$ are the Reynolds stresses (Pa) .

The Reynolds stresses are modelled using the SST $\kappa - \omega$ model^42^. The two transport equations for the turbulent kinetic energy $\kappa$ (m^2^/m^3^) and the turbulent dissipation rate $\omega$ (1/s), are given below.

Closure $$\kappa - \omega$$ equations:

8 $$\begin{aligned} & \frac{\partial {(\rho \kappa )} }{\partial t} + \frac{\partial }{\partial x_j} \left( \rho \kappa \langle u_i \rangle \right) = \frac{\partial }{\partial x_j} \left( \left( \mu + \frac{\mu _t}{\sigma _k} \right) \nabla \kappa \right) + \left( 2 \mu _t S \cdot S - \frac{2}{3} \rho \nabla \kappa \langle u_i \rangle I \right) - \beta ^* \rho \kappa \omega \end{aligned}$$

9 $$\begin{aligned} & \frac{\partial {(\rho \omega )} }{\partial t} +\frac{\partial }{\partial x_i} \left( \rho \omega \langle u_i \rangle \right) = \frac{\partial }{\partial x_j} \left( \left( \mu + \frac{\mu _t}{\sigma _{\omega }} \right) \nabla \omega \right) + \gamma _1 \left( 2 \rho S \cdot S - \frac{2}{3} \rho \omega \langle u_i \rangle \right) - \beta _1 \rho \omega ^2 \end{aligned}$$

Here, $S = \frac{1}{2} \left( \nabla \langle u_i \rangle + {(\nabla \langle u_i \rangle )^T} \right)$ is the strain tensor, $\beta _1$ and $\beta ^*$ are closure constants, $\sigma _{\kappa }$ and $\sigma _{\omega }$ are the turbulent Prandtl number for turbulent kinetic energy, $\kappa$, and specific dissipation rate, $\omega$, respectively. $\mu _t$ is the turbulent viscosity (Pa,s). Menter provided additional information and a detailed explanation of the SST $\kappa - \omega$ model and its model constants^42^. The turbulent transport contributions are modelled via the $\kappa - \omega$ model^42^ in the same way as in our previous work^31^.

Energy balance

10 $$\begin{aligned} \frac{\partial \rho \langle E \rangle }{\partial t} + \frac{\partial }{\partial x_i} \left[ \langle u_i \rangle (\rho \langle E \rangle + \langle p \rangle ) \right] = \frac{\partial }{\partial x_j} \left[ k_{eff} \frac{\partial \langle T \rangle }{\partial x_j} + \langle u_i \rangle (\tau _{ij})_{eff} \right] + S_h \end{aligned}$$

where *E* is the specific total energy (J/kg), $k_{eff}$ represents the effective thermal conductivity (W/m,K), and $(\tau _{ij})_{eff}$ represents the deviatoric stress tensor (Pa), which is used to represent viscous heating. $S_h$ represents the source term (W/m^3^) and includes the heat of reaction term, to be discussed in the kinetics section.

The deviatoric stresses are modelled as:

11 $$\begin{aligned} (\tau _{ij})_{eff} = \mu _{eff} \left( \frac{\partial \langle u_j \rangle }{\partial x_i} + \frac{\partial \langle u_i \rangle }{\partial x_j} \right) -\frac{2}{3} \frac{\partial u_k}{\partial x_k}\delta _{ij} \end{aligned}$$

where $\mu _{eff} = \mu + \mu _t$ is the effective viscosity (Pa,s) and $\delta _{ij}$ is the Kronecker delta.

Transport equation for species *i*

12 $$\begin{aligned} \frac{\partial \rho \langle Y_i \rangle }{\partial t} + \frac{\partial \rho \langle Y_i \rangle \langle u_j \rangle }{\partial x_j} = \frac{\partial }{\partial x_j} \left( \rho D_{eff} \frac{\partial \langle Y_i \rangle }{\partial x_j} \right) + \Sigma _{j = 1}^{nrxn} \nu _{ij} R_j \end{aligned}$$

where $Y_i$ is the mass fraction of species *i* (-), $D_{eff} = D_i + D_t$ is the effective diffusivity (m^2^/s), $D_i$ is the mass diffusivity of species *i* in the mixture (m^2^/s), $D_t$ is the turbulent mass diffusivity (m^2^/s), $\nu _{ij}$ is the stoichiometric coefficient of species *i* in reaction *j*, and $R_j$ is the source term (mol/m^3^,s), the details of which are discussed in the kinetics section.

A porous medium approximation is used to describe the catalyst section of the geometry. Within the porous medium, laminar flow is enforced. The pressure drop due to the flow inside the catalyst is thus due to the viscous losses, and the permeability used is $2.74 \cdot 10 ^{7}$ m^-2^ in the flow direction and $2.74 \cdot 10^{10}$ m^-2^ in the perpendicular directions.

### Initial and boundary conditions

For the transient cases, the inlet boundary conditions are specified as functions of time at the velocity-inlet boundary. Turbulence boundary conditions are determined from an assumed turbulent intensity (5%) and length scale parameters. The turbulence intensity is assumed to follow $I_{turb} = 0.16 {Re}^{- 1/8}$, while the turbulent length scale is set to 7% of the hydraulic diameter of the inlet duct. At the outlet, a pressure-outlet boundary condition with zero gauge pressure is specified. Adiabatic no-slip boundary conditions are applied at the walls.

### Solution methodology

The Semi-Implicit Pressure-Linked Equation (SIMPLE) method is employed to solve for pressure and velocity. The first-order Upwind Scheme is used in discretizing the convective terms for momentum, $\kappa$, $\omega$, and energy. Pressure discretization achieves second-order accuracy, while diffusional terms are discretized with a second-order accurate central differencing scheme. Transient terms are handled with a first-order implicit formulation. Regions of the mesh exhibiting significant gradients are refined as necessary, and this refined grid is thereafter used for obtaining solutions. Iterative convergence is evaluated through scaled residuals, which must decrease by at least three orders of magnitude within the iterations of a time step (or during the duration of a steady-state simulation). Additionally, total mass and energy balances are checked to ensure steady states, and convergence was also monitored by tracking a key property, such as temperature, on a defined plane and on points. In the transient simulations, the time step was chosen so as to maintain a global Courant number below unity.

**Table 3:** Mesh details for grid independence.

Mesh	Total cell count	Difference in area-weighted average velocity [%]
M1	634,669	0.08
M2	700,388	0.01
M3	758,759	0

A mesh independence study was performed in which the sensitivity of solution variables to the mesh resolution was assessed on several meshes. As shown in Table 3, the effects of flow maldistribution were found to be well captured on a mesh of approximately 700,000 cells (mesh M2), which was thereafter used for the simulations.

The complete CFD model has been validated against the experimental results of Pandya *et al.*^38^ for steady-state simulations on the temperature interval 300-600 K in our previous work^31^, where it was shown that the light-off temperature and the peak conversions and trends of CO and $\hbox {C}_3\hbox {H}_{6}$ agree well with the experimental values. Before that, the capability of the CFD model to correctly predict temperature maldistribution under non-reactive conditions has also been validated against experimental data^28^.

### Numerical simulations

Two distinct sets of reactive CFD simulations were conducted: the first involved steady-state reactive simulations at various GHSVs and inlet temperatures, while the second set comprised transient reactive simulations with step changes in temperature and mass flowrates. User defined functions (UDFs) were employed in ANSYS Fluent to specify the reaction rates. In the 1D-SCM model, the ODE solver ode15s is utilized to compute solutions for species balance and energy equations. The inlet concentrations of the species are given in Table 4.

**Table 4:** Species concentrations used in the steady state and transient simulations.

Species	CO	$\hbox {O}_{2}$	$\hbox {CO}_{2}$	$\hbox {C}_3\hbox {H}_{6}$	$\hbox {H}_{2}$	$\hbox {H}_2\hbox {O}$	NO	$\hbox {NO}_{2}$	$\hbox {N}_{2}$
Concentration	100 ppm	6%	10%	350 ppm	33 ppm	10%	150 ppm	0	Remaining

**Fig. 9:**
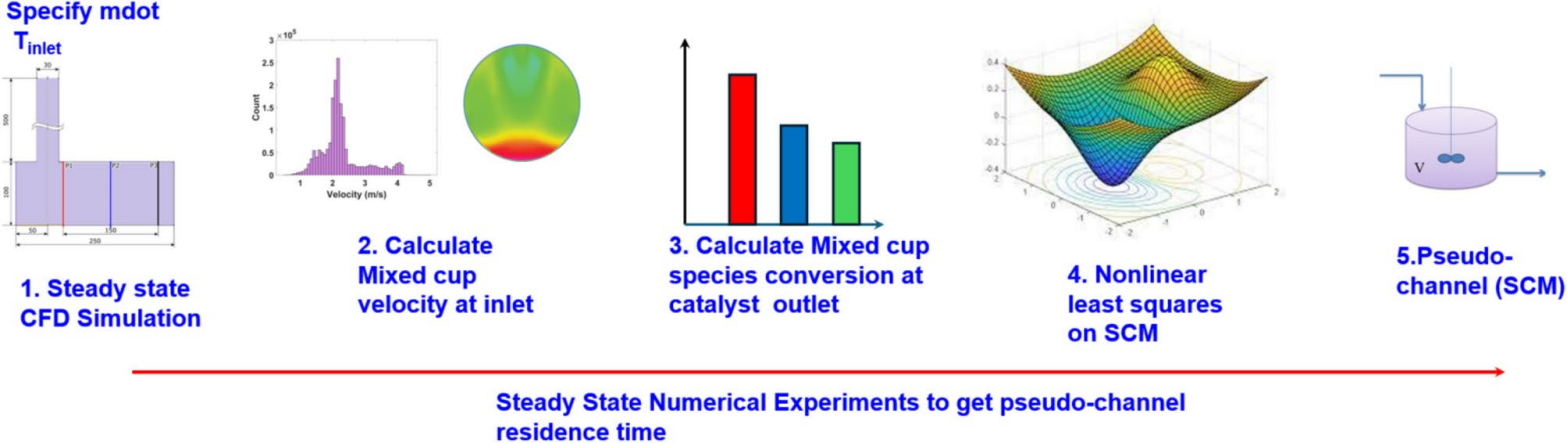
Schematic of steps in the steady state reactive simulation sequence.

Steady state reactive simulations were performed at four GHSVs, namely 40 000, 50 000, 750 000, and 100 000 $\textrm{h}^{-1}$, corresponding to 0.017, 0.018, 0.028 and 0.037 kg/s mass flowrates. Ten temperatures (350 K to 575 K in intervals of 25 K) were used for the inlet temperature. These variations gave 40 sets of operating conditions to obtain the mapped inlet velocity of the corresponding pseudochannel. The values of conversions of the three species namely, CO, $\hbox {C}_3\hbox {H}_{6}$ and NO, at the outlet plane of catalyst were chosen as the desired conversion in the objective function. The lsqnonlin algorithm would then provide the mapped value of SCM inlet velocity at every temperature that would be obtained from an SCM solution. This sequence is shown in Figure 9.

**Fig. 10:**
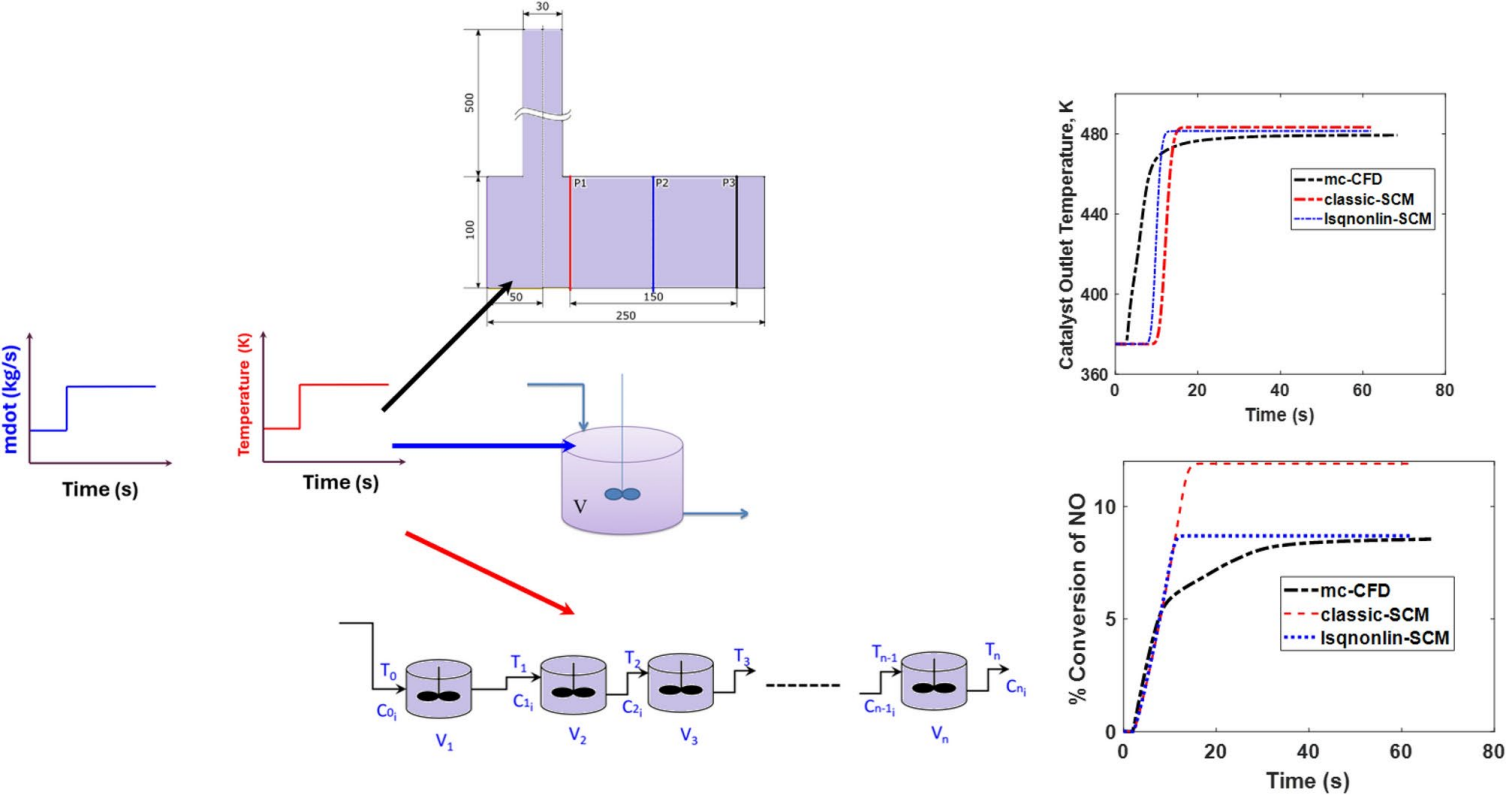
Schematic of steps in the transient reactive simulation sequence.

Two transient simulation variants were performed. The first, termed the velocity transient, is a step change in mass flowrate (velocity) holding temperature constant, and the second, termed the temperature transient, is a step in temperature holding mass flowrate constant. In the former, the mass flowrate steps up from 0.018 kg/s (corresponding to 50 000 $\textrm{h}^{-1}$) to 0.028 kg/s (corresponding to 75 000 $\textrm{h}^{-1}$). In the temperature transient, the two levels of temperature are 375 K and 475 K. The temperature levels are so chosen that CO-light off condition is captured. CFD simulations, SCM simulations with the actual nominal velocity (corresponding to the inlet mass flowrate and temperature) and the pseudochannel velocity are run and the results are compared to assess the performance. This sequence is shown in Figure 10.

## Data Availability

The datasets generated during and/or analysed during the current study are available from the corresponding author on reasonable request.
